# Auditory event-related potentials associated with perceptual reversals of bistable pitch motion

**DOI:** 10.3389/fnhum.2014.00572

**Published:** 2014-08-08

**Authors:** Gray D. Davidson, Michael A. Pitts

**Affiliations:** Department of Psychology, Reed CollegePortland, OR, USA

**Keywords:** bistable perception, auditory awareness, ERP, consciousness

## Abstract

Previous event-related potential (ERP) experiments have consistently identified two components associated with perceptual transitions of bistable visual stimuli, the “reversal negativity” (RN) and the “late positive complex” (LPC). The RN (~200 ms post-stimulus, bilateral occipital-parietal distribution) is thought to reflect transitions between neural representations that form the moment-to-moment contents of conscious perception, while the LPC (~400 ms, central-parietal) is considered an index of post-perceptual processing related to accessing and reporting one’s percept. To explore the generality of these components across sensory modalities, the present experiment utilized a novel bistable auditory stimulus. Pairs of complex tones with ambiguous pitch relationships were presented sequentially while subjects reported whether they perceived the tone pairs as ascending or descending in pitch. ERPs elicited by the tones were compared according to whether perceived pitch motion changed direction or remained the same across successive trials. An auditory reversal negativity (aRN) component was evident at ~170 ms post-stimulus over bilateral fronto-central scalp locations. An auditory LPC component (aLPC) was evident at subsequent latencies (~350 ms, fronto-central distribution). These two components may be auditory analogs of the visual RN and LPC, suggesting functionally equivalent but anatomically distinct processes in auditory vs. visual bistable perception.

## Introduction

Bistable stimuli refer to a class of physically unchanging stimuli that give rise to two mutually exclusive conscious percepts (Necker, 1832; Rubin, 1958; Bool et al., 1981; Lemmo, 2006). The use of bistable stimuli in functional neuroimaging and electrophysiological research allows one to correlate different brain states to different subjective percepts while holding sensory input constant. Neural correlates of bistable perception have been explored for a variety of static visual stimuli (e.g., Başar-Eroglu et al., 1993; Kornmeier and Bach, 2004; Pitts et al., 2007; see Sterzer et al., 2009 for a review), binocular rivalry (e.g., Lansing, 1964; Blake et al., 1992; Tong et al., 1998, 2006; Meng and Tong, 2004; Alpers et al., 2005; Pitts and Britz, 2011), apparent motion stimuli (Sperling et al., 1985; Müller et al., 1999; Kaneoke et al., 2009; Kim et al., 2009; Genç et al., 2011; Strüber et al., 2014), and more recently, auditory streaming stimuli (Gutschalk et al., 2005; Dykstra et al., 2011; Kashino and Kondo, 2012; see Snyder et al., 2012 for a review).

In order to time-lock electrophysiological recordings to stimulus onset, a number of studies have employed intermittent (as opposed to constant) stimulus presentation methods (Leopold et al., 2002; Kornmeier and Bach, 2004, 2005, 2006, 2009, 2012; Kornmeier et al., 2007, 2009, 2014; Pitts et al., 2007, 2008; Britz et al., 2009; Intaitė et al., 2010, 2013, 2014; Ehm et al., 2011; Pitts and Britz, 2011). Intermittent paradigms, particularly those in which each trial consists of a brief stimulus (e.g., ~800 ms stimulus duration) followed by a brief blank interval (e.g., ~400 ms inter-stimulus interval (ISI)), have been shown to constrain perceptual reversals to occur only at stimulus-onset while approximating reversal rates found in constant presentation paradigms, i.e., reversals occur every ~2–6 s (Orbach et al., 1963, 1966; Leopold et al., 2002; Britz et al., 2009; Kornmeier et al., 2009). Subjects are trained to report when a perceptual reversal occurs across adjacent trials or to report their specific percept after each individual trial. In either case, brain activity elicited by each stimulus-onset can be sorted according to whether perception reversed or remained stable relative to the previous trial.

Previous studies that compared event-related potentials (ERPs) for reversal vs. stable trials have consistently reported two components: the “reversal negativity” (RN), a negative-going difference for reversal vs. stable trials over bilateral occipital-parietal scalp regions from ~170–350 ms; and the “late positive complex” (LPC), a positive-going difference between reversal and stable trials over the central-parietal scalp from ~350–600 ms (Başar-Eroglu et al., 1993; Kornmeier and Bach, 2004; Pitts et al., 2007; Britz et al., 2009; Koivisto and Revonsuo, 2010). Based on the estimated locations of their neural generators as well as their timing and sensitivity to top-down and task-based manipulations, it has been suggested that the RN is a neural marker of the transition between the two perceptual representations (but see Intaitė et al., 2010), while the LPC reflects the outcome of this perceptual change, e.g., the updating of working memory required to perform the perceptual-reporting task (Pitts et al., 2008, 2009; Pitts and Britz, 2011).

While a number of experiments have replicated the RN and LPC effects using a variety of stimuli and presentation parameters (e.g., Kornmeier and Bach, 2004; Pitts et al., 2007; Britz et al., 2009; Intaitė et al., 2010), analogous reversal-related components have not yet been identified in sensory modalities other than vision. The most well developed bistable auditory paradigm to date consists of a melodic ABA sequence that can be perceived as two types of continuous patterns: a repeating lo-hi-lo triplet, or two continuous streams at different frequencies (Bregman, 1994; Gutschalk et al., 2005; van Noorden, unpublished). Pressnitzer and Hupé (2006), using the ABA auditory stimulus and a matched visual stimulus, found equivalent distributions of perceptual durations across modalities (short percept durations were common and the frequency of longer stable periods decreased monotonically), while the duration of a given stable period was not correlated to the duration of the previous stable period (suggesting that reversals in both modalities were stochastic). Furthermore, when subjects were instructed to voluntarily maintain a given percept as long as possible, percepts inevitably switched on a comparable number of trials across modalities. Taken together, these findings suggest the existence of auditory equivalents to visually bistable figures, at least in terms of perceptual dynamics (Pressnitzer and Hupé, 2006).

The neural basis of auditory bistability has been explored in a limited number of studies (see Gutschalk and Dykstra, 2014 for a review). Although a wide range of methods and stimuli have been employed (e.g., Sato et al., 2004; Cusack, 2005; Gutschalk et al., 2005; Snyder et al., 2006; Kondo and Kashino, 2007, 2009; Snyder et al., 2009; Dykstra et al., 2011; Schadwinkel and Gutschalk, 2011; Kondo et al., 2012), to our knowledge no previous study has combined the intermittent paradigm with ERPs in the auditory domain. In order to obtain ERPs in an intermittent bistable auditory paradigm comparable to visual paradigms, a novel stimulus is required as the streaming ABA stimulus is not directly amenable to this approach. To create such a stimulus, we began by exploring stimuli with ambiguous pitch characteristics, namely “tritone” stimuli which are derived from Shepard tones.

**Figure 1:**
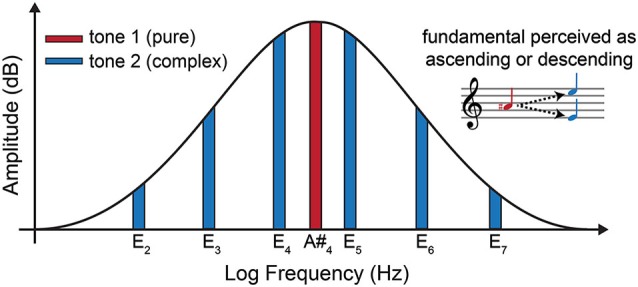
**Frequency spectrum diagram of the first (pure) tone (red) and the second (Shepard) tone (blue) of the bistable tritone stimulus employed in the current experiment**. The horizontal axis is logarithmic reflecting perceptual as opposed to physical distances between frequencies. The musical staff (upper right) shows the two possible perceived pitch movements from tone 1 to tone 2. Due to the pitch ambiguity of tone 2, listeners perceive this pitch as either E_4_ or E_5_, which results in ascending or descending pitch percepts relative to tone 1.

Shepard tones (Shepard, 1964, 1982) are complex auditory stimuli possessing pitch class information but lacking pitch height information (e.g., a Shepard tone might be recognizable as pitch class C# while the specific C# octave is ambiguous). These tones are typically composed of ten octave-related pure-tone harmonics, the amplitudes of which are constrained by a Gaussian envelope as shown in Figure 1. When subjects are asked to make judgments about the direction of pitch motion between paired Shepard tones they inevitably use proximity as the key factor. Thus, a pair of Shepard tones built respectively on pitch classes C# and D will be perceived as ascending in pitch (a distance of 1 semitone) rather than descending (a distance of 11 semitones). When a pair of Shepard tones are exactly six semitones apart, an interval called a tritone, listeners can no longer rely on proximity to make judgments about pitch motion but will still confidently report perceiving either an ascending or descending pitch motion. Moreover, transposition up or down the scale (while maintaining the ambiguous six semitone relationship) leads the same listeners to reverse their pitch motion judgments and different listeners will often hear opposite movements when listening to the same tone pair (Deutsch, 1986). These effects are collectively referred to as the “tritone paradox” and have been extensively investigated by Diana Deutsch et al. (Deutsch, 1987, 1991, 1992, 1994, 1997; Deutsch et al., 1987; Ragozzine and Deutsch, 1994; Repp, 1994, 1997; Giangrande, 1998; Chalikia and Leinfelt, 2000; Chalikia et al., 2000).

Previous investigations of the tritone paradox suggest that each individual possesses a *peak pitch class*, which represents the turning point at which their perception will reverse between ascending and descending pitch motion as the stimulus is transposed around the pitch class circle (Deutsch et al., 1987; Deutsch, 1991, 1994; Ragozzine and Deutsch, 1994; Chalikia and Leinfelt, 2000; Chalikia et al., 2000). For tone pairs starting on this peak pitch class, subjects report nearly equal ascending and descending perceptions across trials. For tone pairs away from the peak pitch class, subjects show a strong bias to perceive a particular pitch motion, but rarely 100%, meaning that for nearly all listeners, any pair of tritone stimuli can be perceived as bistable (Deutsch, 1986, 1987). Indeed preliminary tests showed that with minimal training, most listeners can be taught to hear paired Shepard tones as ascending and descending with equal probability. Tritone stimuli therefore, may be ideally suited to serve as bistable auditory stimuli. Surprisingly, only a few previous studies have noted the bistable characteristics of Shepard tone pairs (Giangrande et al., 2003; Repp and Knoblich, 2007; Repp and Thompson, 2010).

The current investigation utilized a variant of Deutsch’s tritone stimuli to induce bistable perception in the intermittent paradigm. Pairs of tones were constructed such that the first tone was unambiguous (a pure tone) and the second tone was a Shepard tone, six semitones apart from the first (i.e., a tritone). Thus, on any given trial, subjects perceived the pitch motion between the two tones as ascending or descending while the physical properties of the tones remained constant. Behaviorally, we sought to determine whether this particular auditory bistable stimulus exhibits characteristics common for visual bistable figures, e.g., mutual exclusivity, inevitability of reversals, unpredictability of reversals, (Leopold and Logothetis, 1999). We also recorded the EEG, time-locked ERPs to the onset of each tone pair, and compared ERPs according to whether perceived pitch direction changed (reversal) or stayed the same (stable) across adjacent trials. These ERP comparisons allowed us to identify potential auditory analogs to the visual RN and LPC components.

## Methods

### Participants

Twenty-six Reed College students and recent alumni (ages 18–30, 52% female) participated as volunteers. In exchange for participation, subjects were entered into a lottery with a chance to win $150. Written informed consent was obtained from each subject prior to participation in the experiment. No participant reported a history of brain injury or any other neurological condition that might affect his or her electrical brain activity. All procedures adhered to federal regulations and were approved by the Reed College Institutional Review Board. After analysis, five subjects were excluded due to insufficient numbers of trials in at least one condition (less than 100). The final analysis included the remaining 21 participants.

### Stimuli

Each stimulus consisted of a pair of tones: a pure tone at 466.164 Hz. (an A#) followed by a complex Shepard tone built from six octave-related sinusoidal harmonics, each in pitch class E (pitches related perceptually by octaves doubled in frequency: 82.407 Hz, 164.814 Hz, 329.628 Hz, 659.256 Hz, 1318.512 Hz, and 2637.024 Hz). These particular tones were selected because the experiment was to be conducted on the west coast of the United States, and Shepard tone pairs built on A# and E have been shown to be most commonly ambiguous for Californian listeners (Deutsch, 1991). Each tone was presented for 400 ms in immediate succession (no silent interval between tones). Each tone pair was separated by a silent ISI lasting a random duration between 500–900 ms. During each block of trials, a sequence of 65 tone pairs was presented. A spectral diagram of the first and second tones is provided in Figure 1. Details concerning the construction of these tones can be found in Deutsch (1987). The tone pairs employed in the current study differed from Deutsch’s tritone paradox (Deutsch, 1986, 1987) in that the first tone was a pure tone rather than a Shepard tone. This modification confines pitch ambiguity to the second tone, thus allowing ERP analyzes to focus on neural responses to the onset of the second tone. All stimuli were presented at ~80 dB via two Logitech Surround Sound Speakers (model Z906; frequency response: 35 Hz–20 kHz), positioned at equal distances (1.5 m) from the participant’s ears.

### Procedure

All experiments were conducted in a sound-attenuated, electrically shielded booth (Industrial Acoustics Company). While the EEG cap was being prepared and electrical impedances lowered, subjects practiced perceiving the stimuli until they reported fluency of judgment between ascending and descending perceptions. Because previous studies have found that reversal rates increase during the initial trials of an experiment with novel bistable stimuli (Long and Toppino, 2004), each participant was exposed to a minimum of two blocks of stimuli (130 total) before EEG recording began.

Participants maintained fixation on a small, centrally located fixation cross (subtended angle of 0.76°) which was visible throughout all stimulus presentations and ISIs. Subjects were trained to report their perception after each stimulus pair by pressing one of two buttons with the index or middle finger of the right hand, indicating ascending or descending pitch perception (buttons counterbalanced across participants). In the case that a tone pair sounded ambiguous with respect to direction of pitch motion, participants were instructed to refrain from pressing any buttons. The time window for viable responses was set to 0–1300 ms after tone 1 onset and any trials in which subjects’ responses fell outside this window were excluded.

**Figure 2:**
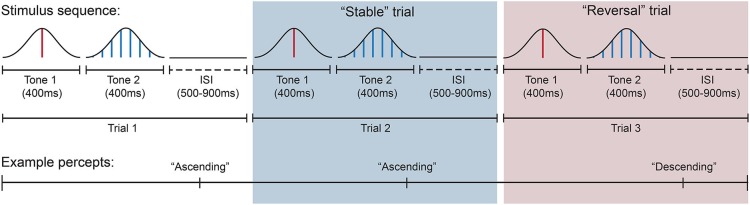
**Intermittent stimulus presentation sequence and corresponding example percepts across trials**. In this example, the subject reports the perceived pitch direction of the tone pair as ascending in the first trial (white background) and also ascending in the second trial (blue background). Thus the second trial would be categorized as a “stable” trial because perceived pitch direction remained the same across successive trials. The tone pair in the third trial (red background) was perceived as descending and would be categorized as a reversal trial because perceived pitch direction differed from the immediately preceding trial. Each tone was presented for 400 ms in immediate succession, while trials were separated by a silent ISI of variable duration (500–900 ms). Subjects indicated their perceived pitch direction after each tone pair.

Trials were presented in blocks of 65, separated by short rest breaks of approximately 15 s. Sets of four blocks were separated by longer 2-min breaks, and each subject completed 12 or 16 blocks for a total of 780 or 1040 total trials. Trials were segregated into conditions by comparing the reported perception on a given trial to the perception on the previous trial. A trial was considered a *reversal* if reported percepts differed between adjacent trials and *stable* if perception remained the same. The experiment commenced after 12 blocks (3 sets of 4) if each of these two conditions (reversal and stable) contained at least 150 trials; otherwise an additional set of four blocks was administered. Figure 2 shows a schematic of the stimulus presentation sequence and perceptual reporting task.

### EEG/ERP methods

EEG scalp voltages were recorded using a 96-channel electrode cap with equidistant electrode placements (EasyCap). Signals were amplified via 3 × 32 BrainAmp Standard amplifiers (Brain Products), bandpass filtered between 0.1 and 150 Hz, and digitized at 500 Hz. During data collection all channels were referenced to CPZ and were re-referenced to the average of the left and right mastoids offline. Eye position and eye movements were monitored via a vertical electrooculogram (EOG) channel positioned under the left eye and left and right horizontal EOG channels (re-referenced to each other offline to form a bipolar pair). All sensors were individually adjusted until the impedance of each was less than 5 kΩ.

ERPs were time-locked to the onset of the first stimulus, and separated into reversal and stable conditions as described above. Trials with eye movement, blink, or muscle artifacts within a time window −100 ms to +1000 ms (relative to tone 1) were detected and rejected semi-automatically by a combination of computer-based peak-to-peak amplitude thresholds and visual inspection. Trials in each condition were averaged, low-pass filtered at 30 Hz, and baseline corrected from −100 to 0 ms prior to tone 1 onset. Because pitch ambiguity occurred only for the second tone, the timing of all ERP effects are reported with respect to the onset of tone 2 (hereafter, time-zero = tone-2-onset).

Repeated measures analysis of variance (ANOVA) were carried out on mean amplitudes within latency windows corresponding to amplitude differences observed in the grand average ERPs for the reversal and stable conditions. An auditory reversal negativity (aRN) was apparent from 120–220 ms, bilaterally over fronto-central electrode sites 9, 10, 22, 23, 24, 38, 39, 21, 37 and 17, 18, 32, 33, 34, 51, 52, 35, 53 (corresponding to electrodes C4, C6, FC4, FC6, F2, F4, F6, AF4 and C3, C5, FC3, FC5, F1, F3, F5, and AF3 of the International 10/20 system), and was assessed via 2 × 2 × 9 ANOVA with the factors perception (reversal vs. stable), hemisphere (left vs. right), and electrode (channels listed above). An auditory late positive complex (aLPC) was evident at subsequent latencies (320–380 ms) at electrodes 2, 3, 6, 7, 8, 19, 20 (corresponding to C1, C2, FCZ, F1, FZ, F2 and AFZ), and was assessed by a 2 × 7 ANOVA with the factors perception and electrode. Exploratory analyzes were conducted in a third ANOVA on a difference component that was evident just prior to the onset of tone 2, from −80 to −20 ms, over fronto-central electrodes 2, 7, 8, 19, and 20 (corresponding to FCZ, F1, FZ, F2 and AFZ).

## Results

### Behavioral results

Overall, participants reported perceiving ascending pitch motion on 51.2% of all trials, descending pitch motion on 45.84% of trials, and ambiguous or unclear percepts on 2.96% of trials. These results suggest mutual exclusivity of percepts elicited by this bistable stimulus. Individual perceptual biases varied in both directions, with one subject reporting ascending vs. descending pitch on 39% and 61% of trials, respectively, and another reporting the same percepts with an opposite bias: 60% ascending and 40% descending.

**Figure 3:**
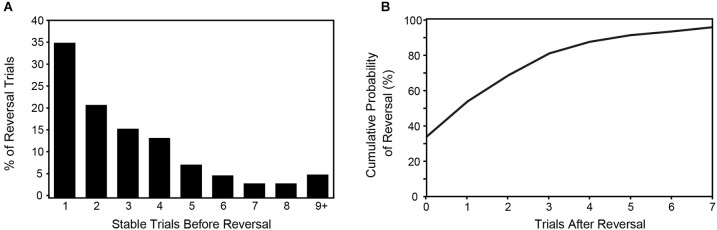
**Behavioral data showing the number of stable trials preceding a reversal trial as a function of the overall frequency of reversal trials (A), and the cumulative probability of a reversal occurring as a function of trials following a reversal (B)**. Both figures demonstrate the inevitability of perceptual reversals over time, a key feature of bistable stimuli.

Perceptual reversals of pitch motion occurred on average every 2.92 trials (4.38 s), and followed a monotonically decreasing distribution (Figure 3), meaning the probability of a stable period continuing decreased steadily on each trial. An exponential decay function fitted to these data points matched closely (*r*^2^ = 0.98). The shape of this curve indicates that reversals were inevitable since the probability that a reversal will *not* occur decreased asymptotically to zero. Reversals were also shown to be unpredictable, meaning that a given reversal could not be predicted by perceptual reports on preceding trials. In particular, the correlation between the lengths of adjacent stable periods (as measured by number of stable perceptions before a reversal) was low (*r* = 0.147). Mean reaction time, collapsed across both conditions was 838.6 ms post-tone-2-onset (sd = 150.0), and did not differ statistically between ascending and descending percepts (*p* = 0.945) or between stable and reversal trials (*p* = 0.701).

### ERP results

For ERP analyses, the overall percentage of trials in each condition was 65.42% stable and 34.58% reversal. Minimum trials per subject for each condition were 186 stable and 102 reversal. Mean number of trials per condition across subjects was 389 (sd = 153) stable and 201 (sd = 84) reversal.

A negative-going amplitude difference for reversal trials (aRN) was evident from 120–220 ms (post-tone-2-onset) over fronto-central scalp locations, *F*_(1,20)_ = 15.2, *p* < 0.001, partial eta-squared = 0.43 (mean amplitudes: reversal = −2.8 μV (sem = 0.53); stable = −2.0 μV (sem = 0.58)). This component was present in both hemispheres with a clear bilateral distribution. The hemisphere × condition interaction was not significant, *F*_(1,20)_ = 0.017, *p* = 0.9. No other effects or interactions approached significance.

**Figure 4:**
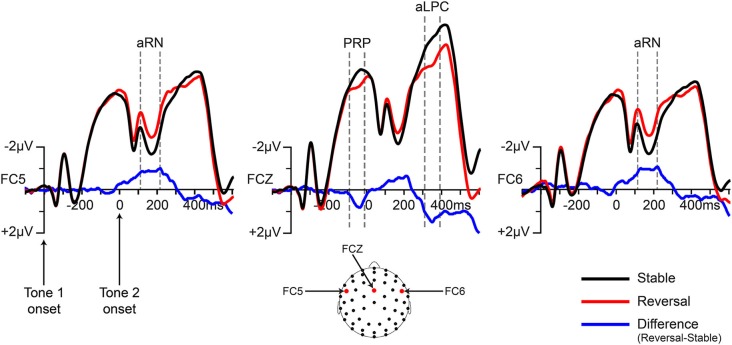
**Grand-averaged ERPs for stable and reversal trials and difference waves (reversal minus stable) at electrodes representative of the observed amplitude differences**. ERPs were time-locked to tone 1, but note that the time scale is adjusted such that tone-2-onset was treated as time zero for analysis purposes (because tone 2 was the ambiguous tone). The time windows of the main components of interest are denoted by the dotted gray lines (see main text for details): aRN = auditory reversal negativity; aLPC = auditory late positive complex; PRP = pre-reversal positivity.

**Figure 5:**
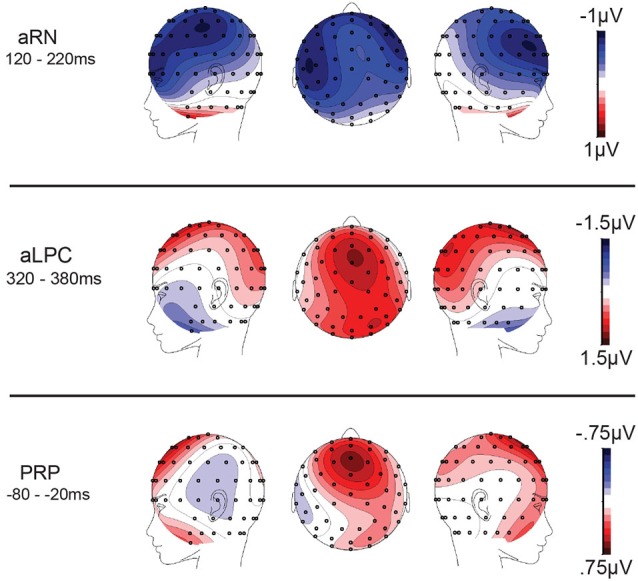
**Grand-averaged difference wave topographies (reversal minus stable) for each of the components of interest, averaged across the indicated time windows**. As in Figure 4; time zero refers to tone-2-onset.

A subsequent, positive-going difference for reversal trials (aLPC) was apparent from 320–380 ms (post-tone-2-onset) with a midline distribution over the fronto-central scalp, *F*_(1,20)_ = 6.2, *p* = 0.02, partial eta-squared = 0.24 (mean amplitudes: reversal = −6.0 μV (sem = 0.62); stable = −6.9 μV (sem = 0.71)). ERPs from the reversal and stable conditions at electrode sites centered on the aRN and aLPC components are shown in Figure 4. Difference waves were computed by subtracting stable ERPs from reversal ERPs. Scalp topographies of these difference waves are provided in Figure 5.

An unexpected ERP difference was observed during a time window immediately prior to the onset of tone 2 (−80 to −20 ms). The paired presentation sequence, with an unambiguous tone followed by an ambiguous tone, allowed for investigation of brain activity preceding perceptual reversals, i.e., during the time window between tone-1-onset and tone-2-onset. The pre-reversal ERP difference observed here had a very similar scalp distribution to the aLPC and was tested over similar electrode sites. This difference trended towards statistical significance, *F*_(1,20)_ = 2.93, *p* = 0.1, partial eta-squared = 0.13 (mean amplitudes: reversal = −4.9 μV (sem = 0.51); stable = −5.4 μV (sem = 0.57)), and should be investigated in future experiments that utilize paired bistable stimuli. We refer to this difference component here as the pre-reversal positivity (PRP).

Finally, we compared ERPs elicited by the bistable tritone stimuli according to whether subjects perceived ascending vs. descending pitch motion (i.e., percept A vs. B instead of reversal vs. stable). This comparison revealed no amplitude differences at any time point, for any electrode on the scalp. While it is reasonable to assume that there must be some difference in neural activity underlying the two perceptual states, scalp ERP measures were unable to resolve this difference.

## Discussion

The goals of this study were to create a bistable auditory stimulus suitable for use with the intermittent ERP paradigm, test the perceptual dynamics of this stimulus, and identify potential auditory analogs of the RN and LPC components commonly found for visual bistable figures. The physical features of the tritone stimulus remained constant throughout the experiment while subjects reported their alternating perceptions of pitch motion (ascending or descending pitch) after each trial. Behaviorally, we found that this auditory bistable stimulus shared similar qualities to visual bistable figures, including mutual exclusivity of the two alternative percepts, inevitability of perceptual reversals over time, and unpredictability of reversals for any given trial. The ERP results revealed two difference components at similar (slightly earlier) latencies as the visual RN and LPC, but with more frontal (compared to posterior) scalp distributions. We provisionally refer to these two difference components as the aRN and aLPC.

### Perceptual dynamics of the bistable tritone stimulus

Our behavioral results showed that for this particular derivation of the tritone stimulus, reversals typically occurred after a small number of trials (every ~3 trials or ~4.5 s). We further found that reversal intervals decreased monotonically, indicating that on a long enough timescale the probability that a stable period will continue decreases to zero and therefore reversals are inevitable. The two alternative percepts were reported with near equal probability (51% vs. 46%) while ambiguous or otherwise unclear percepts were only reported on 3% of trials. This finding confirms the mutual exclusivity of percepts elicited by the tritone stimulus. Finally, on any given trial, reversals were found to be nearly stochastic, with a very low correlation between the lengths of adjacent stable periods, and thus very low predictability for the timing of reversals. Randomness of reversals is a property commonly attributed to visual bistable figures. Overall, these results suggest that the perceptual dynamics of the tritone stimulus employed here are similar to those found for most visual bistable stimuli (Leopold and Logothetis, 1999; Leopold et al., 2002; Long and Toppino, 2004; Pressnitzer and Hupé, 2006; Sterzer et al., 2009).

### Auditory vs. visual ERP components associated with perceptual reversals

In the visual domain, two components have been widely reported for perceptual reversals of bistable figures, the RN and LPC. In the current study, potential auditory counterparts to the visual RN and LPC components were identified. The aRN, reported in this experiment was elicited earlier than the visual RN: 120–220 ms post-tone-2 in the current study, relative to ~180–280 ms in previous visual studies. The latency of the aLPC was similarly reduced (320–380 ms post-tone-2) compared to the visual LPC (350–600 ms). Reduced latencies are generally expected for auditory vs. visual ERPs, as the earliest scalp ERP in the auditory modality, the P1 at 20–50 ms, precedes the earliest visual ERP, the C1 at 50–100 ms (Davis, 1939; Spehlmann, 1965; Hillyard et al., 1973; Näätänen and Picton, 1987; Clark et al., 1994).

In addition to latency differences between modalities, the aRN and aLPC showed more frontally-focused scalp distributions compared to the visual RN and LPC reported previously. The aRN was strongest bilaterally at fronto-central locations while the visual RN is typically restricted to parietal and occipital sites (Pitts et al., 2007, 2008; but see Intaitė et al., 2010; Kornmeier and Bach, 2012). Similar to the visual RN, the aRN temporally overlaps the N1/P2 but does not appear to reflect a modulation of the N1 or P2 peaks as evidenced by marked differences in scalp topography, i.e., the N1 and P2 peaks showed a broad central or fronto-central distribution centered on the midline, while the aRN was clearly bilateral. The aLPC was centered on the midline at fronto-central electrode sites compared to the visual LPC which, although widespread, generally centers on parietal and parieto-central electrodes (Kornmeier and Bach, 2004; Pitts et al., 2008, 2009). It will be worthwhile for future studies to estimate the locations of the neuroanatomical sources of the aRN and aLPC, as both components can be isolated via difference waves while the physical stimulus remains identical across the two conditions.

It has previously been suggested that the visual RN reflects the process of updating the contents of consciousness in higher-level (ventral stream) visual areas (Pitts and Britz, 2011). The existence of a reversal ERP component in a second modality, sharing similar timing and polarity, while differing in ways which are expected due to characteristic differences between the two perceptual systems, supports the view that the visual RN and aRN reflect common perceptual processes across modalities. However, the current study as well as previous visual studies, have not yet conclusively demonstrated a link between the RN component and the transition between contents of conscious perception.

Intaitė et al. (2010) argue that the RN does not reflect changes in conscious perception because in one of their conditions, exogenous reversals of unambiguous Necker cubes did not elicit an RN. It is justifiably assumed that any correlate of conscious perceptual change found for endogenous reversals of ambiguous stimuli should also be apparent for exogenous reversals of unambiguous stimulus variants. Kornmeier and Bach (2004), however, reported an RN component for exogenous reversals of unambiguous Necker cubes, and interestingly this exogenous RN was found to occur at earlier latencies compared to the endogenous RN. An interesting extension of the current experiment would be to measure ERPs elicited by both ambiguous tritone stimuli and unambiguous variants in which reversals in pitch perception are controlled exogenously. If the aRN, like the visual RN, indeed reflects changes in conscious content, it should also be found for exogenous reversals of pitch motion, perhaps at slightly earlier latencies.

### Relationship between the aRN and other auditory ERP components

An alternative interpretation of the visual and auditory RN components is that they both reflect shifts in attention that immediately precede or follow the establishment of new perceptual content. Intaitė et al. (2010) employed bilateral displays to test whether the RN is simply an N2pc component in disguise. The N2pc is a well-studied ERP component that reflects shifts in visual spatial attention (Luck and Hillyard, 1994; Hickey et al., 2006; Robitaille and Jolicoeur, 2006; Luck, 2012). An auditory equivalent to the N2pc, labeled the N2ac, has recently been discovered and is thought to reflect similar attention shifts in the auditory domain (Gamble and Luck, 2011). Intaitė et al. (2010), however, found that the RN was distinct from the N2pc component suggesting that the RN reflects some other process besides the shifting of visual spatial attention. One hypothesis is that a transition in object-based attention is a necessary prerequisite for perceptual reversals and that the RN reflects this non-spatial type of attention shift. Alternatively, perceptual reversals might *attract* object-based attentional resources. In this case, the attention shift (and the RN) would be considered a consequence of conscious perceptual change. Currently, the relationship between attention and conscious perception is an open question and a topic of intense debate (Tsuchiya and van Boxtel, 2013). It will be important for future studies to try to tease apart whether the RN and aRN are more closely associated with transitions in conscious perceptual content or with preceding/consequential attentional shifts.

A separate concern with the current aRN results is that this ERP difference component might reflect auditory-change detection more generally. The mismatch negativity (MMN), a well-known auditory ERP component with similar timing, polarity, and scalp distribution as the aRN, is typically elicited by deviant (less probable) stimuli within a sequence of standard (more probable) stimuli (Näätänen et al., 1978, 2004). In the present experiment, participants perceived the tritone stimuli as ascending or descending in pitch with near equal probability (51.2% Ascending vs. 45.8% Descending), but perceptual reversals of pitch motion were experienced far less often than perceptual stability (65.42% stable vs. 34.58% reversal), thus rendering reversal trials as a perceptual “deviant” and stable trials as the “standard”. Because reversal rates varied across subjects, we were able to test whether the aRN and the MMN are indeed the same component by comparing individual subject aRN amplitudes with individual reversal rates. It has commonly been found that decreasing the probability of the deviant stimulus increases the amplitude of the MMN (Näätänen and Kreegipuu, 2012). Thus, if the aRN is identical to the MNN, larger amplitudes would be expected for subjects in which reversals were rarer and smaller amplitudes for subjects in which reversal and stable trials were more equivalent. This analysis, however, did not reveal even a modest relationship between aRN amplitude and reversal probability (*r* = 0.094, *p* = 0.69), suggesting that the aRN and MMN are most likely distinct components.

A third component that the aRN might relate to is the awareness related negativity (ARN), reported in previous auditory masking experiments (Gutschalk et al., 2008; Königs and Gutschalk, 2012; Wiegand and Gutschalk, 2012). The ARN, a negative-going component from ~100–250 ms, has been found to uniquely index detected tones (compared to undetected, physically identical tones) when the target tones are presented within a complex multi-tone background (Gutschalk and Dykstra, 2014). While Gutschalk and colleague’s methods did not include intermittent bistable stimuli, nor did the present investigation make aware vs. unaware comparisons, it is possible that changes in auditory awareness is the common underlying factor across these studies. A visual analog of the ARN, the visual awareness negativity (VAN), has been reported, (Koivisto and Revonsuo, 2003, 2008; Ojanen et al., 2003; Pitts et al., 2012; Pitts and Martinez, 2014; see Railo et al., 2011 for a review), but only one attempt has been made thus far to compare the visual RN to the VAN (Intaitė et al., 2010). Intaitė et al. (2010) concluded that the visual RN and the VAN are unlikely to be the same component, although further research is necessary to verify this claim. In the auditory domain, the relationship between the aRN and ARN remains an open question.

### Does the aLPC reflect post-perceptual processing?

The visual LPC (or simply the LP) has been associated with bistable perceptual reversals for more than 20 years and is visible in both intermittent stimulus-locked as well as continuous response-locked paradigms (Başar-Eroglu et al., 1993). Since its discovery, the LPC has been difficult to distinguish from the P300 (P3b) component, which is thought to index post-perceptual updates to working memory (Donchin and Coles, 1988; Picton, 1992; McEvoy et al., 1998). Whether the task is to report perceptual reversals or to report one’s percept after each trial, working memory is likely utilized for perceptual reporting purposes. Interestingly, although the onset of the visual LPC has been consistent across studies (~350 ms), its duration varies considerably according to the task. For example, Pitts et al. (2008) report an LPC lasting from ~350 to beyond 700 ms when subjects were tasked with reporting reversals of the Necker cube, while Pitts et al. (2009) observed a shorter-duration LPC (~350–600 ms) when the task was simply to report one’s percept after each trial. Furthermore, Kornmeier and Bach (2004) manipulated the task such that subjects reported reversals on some blocks and stability on other blocks. They found a reversal-vs-stable amplitude difference in the LPC time-window regardless of task, but the overall amplitude of the LPC was larger when responses (vs. non-responses) to reversals were required. Overall, these findings suggest some degree of functional overlap between the LPC and the P300, in addition to similarities in polarity, timing, and scalp distribution.

If the LPC and the P300 (specifically the P3b) are identical, one might expect similar scalp topographies across visual and auditory modalities as the P3b has been found to maintain a consistent parieto-central distribution in both visual and auditory target detection tasks (Comerchero and Polich, 1999; Polich, 2007). In the current study, however, the auditory LPC was focused over more anterior scalp regions compared to the visual LPC. If the aLPC and visual LPC reflect the same underlying process, the distinct scalp distribution of the aLPC suggests an incomplete functional overlap with the P3b. To explore this issue further, we performed a correlational analysis between reversal probability and LPC amplitude, as the P3b (similar to the MMN) is known to increase in amplitude for more deviant (less probable) stimuli (Polich, 2012). Interestingly, there was a trend towards a relationship between individual subject reversal rates and aLPC amplitudes (*r* = −0.39, *p* = 0.08), meaning that subjects who experienced perceptual reversals less frequently tended to show larger aLPC amplitudes. While this result does not definitively link the aLPC (and LPC) with the P3b, it suggests at least a partial functional overlap due to its apparent sensitivity to percept probability.

### Pre-reversal positivity and the paired stimulus approach

When ERPs were time-locked to the first tone in the pair which was unambiguous and reversal trials were compared to stable trials, a small positivity was apparent just prior to the onset of the second (ambiguous) tone, 320–380 ms post-tone-1 (20–80 ms pre-tone-2). The timing of this PRP, on the cusp of a changing bistable percept, leaves open the possibility that it may reflect events that contribute causally to perceptual reversals. Pre-stimulus components, predictive of upcoming perceptual reversals have been reported in the past, both for the Necker cube (Britz et al., 2009; Intaitė et al., 2014) as well as for binocular rivalry (Britz et al., 2011), although the functional role of such pre-reversal activity is currently unclear.

The timing of the PRP observed in the current study relates to the onset of the first tone exactly as the timing of the LPC relates to the onset of the second tone (320–380 ms post-tone-onset). The topography of the PRP was also highly similar to the topography of the LPC. It is possible that the PRP and LPC represent a similar process, perhaps related to working memory. A second possibility is that pre-reversal components reflect volition or the intention to switch percepts (or attentional processes related to voluntary reversals). Subjects in the present experiment were instructed to maintain a passive approach to reversals letting them occur without voluntary control, however, it is difficult to ensure a complete lack of intentional influence over reversals in passive viewing situations. While the current study was aimed at measuring perceptual dynamics and ERPs elicited by a novel bistable auditory stimulus, future investigations will benefit from designs and analysis approaches which allow further testing in the pre-stimulus interval.

## Conclusion

This investigation sought to expand the body of research on visual bistability into the auditory domain by pioneering a novel bistable auditory stimulus for use with the intermittent ERP paradigm. The tritone stimulus employed here successfully elicited bistable perceptions, the statistical characteristics of which were similar to those found for visual bistable figures. ERP comparisons between reversal and stable trials revealed potential auditory analogs to the previously reported RN and LPC components. These auditory components, provisionally referred to here as the aRN and aLPC, occurred slightly earlier in time and with more anterior scalp distributions compared to their visual counterparts. While the exact neuropsychological processes contributing to these ERP components remain to be specified, the current results suggest functionally-equivalent yet neuroanatomically-distinct mechanisms underlying auditory and visual bistable perception.

## Conflict of interest statement

The authors declare that the research was conducted in the absence of any commercial or financial relationships that could be construed as a potential conflict of interest.
